# 2508: The role of platelet factor-4 (PF4 or CXCL4) in B cell differentiation

**DOI:** 10.1017/cts.2017.52

**Published:** 2018-05-10

**Authors:** Sara Blick, Craig Morrell, Sara Ture, David J. Field

**Affiliations:** Rochester Institute of Technology, Rochester, NY, USA

## Abstract

OBJECTIVES/SPECIFIC AIMS: To investigate the role of platelet factor-4 (PF4) in B cell differentiation and develop strategies to better modulate B cell differentiation in vitro and in vivo. METHODS/STUDY POPULATION: We use tissue culture and flow cytometry to examine the role of PF4 in B cell differentiation. We use wild type (WT) and PF4^−/−^ mice on a C57Bl6/J background. PF4^−/−^ mice have reduced in vivo B cell differentiation responses. RESULTS/ANTICIPATED RESULTS: We anticipate that our studies will demonstrate that PF4 promotes B cell differentiation in the bone marrow microenvironment. DISCUSSION/SIGNIFICANCE OF IMPACT: The significance of this project may be valuable in developing efficient methods and strategies to increase or limit B cell numbers in vitro and in human disease.

